# Correspondence

**Published:** 1864-06

**Authors:** 

**Affiliations:** Chicago


CHICAGO, May 16, 1864.

DEAR DOCTOR:—Having for a long time promised you an article for the DENTAL REGISTER, and having done nothing very remarkable or worthy of notice myself, I send you the following very interesting case, brought to my notice by Thos. Bevan, M. D., of this city.

The patient's symptoms up to the time when he was first brought to me were described as follows: July 8, 1863, Mr. L. S., merchant, aged 32 years, came to Dr. B., complaining of pain in the right side of the face, extending from the angle of the inferior maxillary to about the middle of the body of the bone. The pain was lancinating in character, coming on in accesses, commencing with a sharp twinge, continuing a few seconds of a most excruciating intensity, then subsiding into a dull aching heavy pain. These periods of attack varied exceedingly both in duration and intensity, lasting from ten seconds to three or four minutes. The repetition of the attacks, as regards frequency, was irregular at times, one only occurring in twenty-four hours; but there was no regular periodicity. No swelling existed, and there was no marked sensitiveness to pressure on any point of the jaw. Certain movements of the jaw in mastication would, however, bring on an attack; yet, by carefully guarding the exercise of the function from violent or grinding motion of the member, he could eat without inducing a return of the attack. The pain continued fixed in this locality for about six weeks, leading his medical adviser to suspect caries of a tooth, or some of the osseous parts in the neighborhood of a nervous filament, and he therefore brought him to me.

On examination I found the teeth free from inflammation or discoverable lesion, the gums healthy, the buccal soft parts normal, and percussion on the teeth caused no pain. There seeming to be nothing that I could do to alleviate his case, and thinking that his malady must yield in time to constitutional treatment, I dismissed him again to the care of his medical attendant.

The seat of the pain baffled discovery; it now would radiate along the side of the face, upwards to the buccal nerve and backwards, leaving decided morbid sensitiveness, extending along the integument to a point over the gasserian ganglion; also, some directions of pressure discovered sensitiveness along the lower curvature of the edge of the malar bone. From this time to the 8th of March, 1864, the pain would fly from point to point of the side of the face, seeming most intense in the superior maxillary and its branches. The attacks became very frequent and distressing, rendering the patient nervous, dyspeptic and utterly miserable. During this period of eight months, quinine, opium and its preparations, alteratives, iron and general reconstituents, hyoscyamus, belladonna, chloroform, aconite, veratrum viride and an exhaustive course of all the agents usually employed in the treatment of Tic were essayed in vain. The patient became worse under them all. Morphine, pushed to decided narcotism, would sometimes give temporary relief, at the expense of headaches, torpid bowels and loss of appetite.

About the first of March he came to me again, and as there seemed to be a little sensitiveness about the right superior wisdom tooth, and as it was useless, having no antagonist, I removed it, hoping it might possibly bring relief. This failed, however, to give any relief, and by my advice and with the consent of his attending physician, he put himself under the care of Dr. Hayes for treatment, by means of the Electro-thermal bath. He took two baths a day for eight days, but without favorable results. Indeed, the Electro-thermal treatment seemed rather to aggravate his symptoms.

His attendant physician, Dr. B., then adopted as a dernier resort, the following treatment, which proved successful. He made a prescription for eighty pills of a proved extract of belladona with oxide of zinc, so that the patient should get $\frac{1}{4}$ grain of belladona with two grains of zinc four times a day in each pill, the dose to be increased one pill each day until six pills were taken at a dose four times in twenty-four hours. He determined to push this to the utmost tolerance of the drug compatible with the safety of the patient. The eighth or ninth day of the full dose as above stated, the patient began to experience decided relief; the attacks were less frequent and severe, until finally they ceased, and have not returned since (May 10, 1864). The drug produced no untoward symptoms beyond dilatation of the pupil and consequent dimness of vision, dryness of the throat, &c., which readily passed off with returning health.

ALLPORT.

